# The effect of age on the incidence of COVID-19 complications: a systematic review and meta-analysis

**DOI:** 10.1186/s13643-021-01636-2

**Published:** 2021-03-20

**Authors:** Sofonyas Abebaw Tiruneh, Zemenu Tadese Tesema, Melkalem Mamuye Azanaw, Dessie Abebaw Angaw

**Affiliations:** 1 Department of Public Health, College of Health Sciences, Debre Tabor University, Debre Tabor, Ethiopia; 2 Department of Epidemiology and Biostatistics, Institute of Public Health, College of Medicine and Health Sciences, University of Gondar, Gondar, Ethiopia

**Keywords:** COVID-19, Age, Acute respiratory distress syndrome, Acute kidney injury, Acute cardiac injury, Shock, Meta-analysis

## Abstract

**Background:**

The coronavirus (COVID-19) pandemic was reported from Wuhan, China, on December 31, 2019, and the pandemic was spread to more than 212 countries in the globe. This meta-analysis aimed to assess the pooled incidence of COVID-19 complications and to identify the association between the incidence of complications and age.

**Methods:**

Comprehensive databases, PubMed, Hinari, and Google Scholar, were used to locate potential articles for this review. Data were extracted using Microsoft Excel and imported to the STATA/MP version 16.0 software for analysis. Heterogeneity between studies was assessed using the Cochrane *Q* test statistics and *I*^2^ test, and small study effect was checked using Egger’s statistical test at 5% significant level. Sensitivity analysis was checked. A random-effects model was conducted to estimate the pooled incidence of COVID-19 complications. Univariate meta-regression was conducted to identify the association between the mean ages with each complication.

**Results:**

From the total of 1237 studies, 12 studies were included with a total of 3064 COVID-19 patients. The most complications were acute respiratory distress syndrome (30.93%, 95%CI 21.3–40.6%) followed by acute liver injury (22.8%, 95%CI 14–31.5%), shock (10.9%, 95%CI 7.4–14.4%), acute kidney injury (7%, 95%CI 3.8–10.4%), and acute cardiac injury (6.4%, 95%CI 2.8–15.6%). Univariate meta-regression revealed that as the mean age increased by 1 year, the incidence of acute respiratory distress syndrome, acute kidney injury, acute cardiac injury, and shock increased by a factor of 2.9 (*β* = 2.9, 95%CI 2.4–3.4, adjusted *R*^2^ = 88), 0.4 (*β* = 0.4, 95%CI 0.04–0.72, adjusted *R*^2^ = 54), 1.6 (*β* = 1.6, 95%CI 1.1–2.1, adjusted *R*^2^ = 85), and 1.1 (*β* = 1.1, 95%CI 0.8–1.5, adjusted *R*^2^ = 26) times respectively.

**Conclusion:**

Significant complications of COVID-19 viral infections were reported. Older populations were a high-risk group of developing adverse complications as compared to their counterparts. Health care professionals should give primary attention to those risk group individuals.

**Supplementary Information:**

The online version contains supplementary material available at 10.1186/s13643-021-01636-2.

## Background

Coronavirus is a large family of viruses that usually causes mild to moderate upper respiratory tract infections. However, three new coronavirus families namely SARS-CoV, MERS-CoV, and SARS-CoV-2 have emerged from animal reservoirs over the past two decades to cause serious and prevalent human illness and death [1]. The recent emerging COVID-19 novel coronavirus is a newly emerging zoonotic public health challenge that emerged at the end of December 31, 2019, in Wuhan, Hubei province of China with a reported cluster of 27 cases of pneumonia of unknown cause. The Coronavirinae family consists of four genera, *Alphacoronavirus*, *Betacorona* virus, *Gammacorona* virus, and *Deltacorona* virus. Based on genetic properties, COVID-19 belongs to the genus *Betacorona* virus [2]. Fever, cough, and dyspnea were the most prevalent clinical presentation of the newly emerging virus infection [3–6].

According to World Health Organization (WHO) report on 26 April 2020, globally, more than 2.8 million cases were confirmed to have COVID-19, and 193,722 (case fatality rate is 6.9%) related deaths have been reported to date; around 48% of cases reported from European countries. The lowest cases of COVID-19 were reported from the Africa Region which is 20,316 confirmed cases (case fatality rate is 4%) [7].

According to Yang et al. report, from the total of 52 hospitalized COVID-19 patients, 67%, 29%, 29%, and 23% of them experienced adverse complications of acute respiratory distress syndrome (ARDS), acute kidney injury (AKI), liver dysfunction, and acute cardiac injury (ACI), respectively. Of patients developing ARDS, AKI, ACI, and liver dysfunction adverse complications, 74%, 80%, 75%, and 60% of them died, respectively [8]. This suggests that experiencing adverse complications has a high risk of COVID-19 mortality.

Documenting the pooled incidence of adverse complications of COVID-19 infection will give valuable information for the timely intervention of the patients. However, more pieces of evidence are needed to know the pooled incidence of adverse complications of patients with COVID-19 as well as the effects of age on the incidence of each complication.

Therefore, this systematic review and meta-analysis will give the pooled incidence of adverse complications among COVID-19 patients. Also, this review also assesses the effects of age on the incidence of each complication.

## Methods

### Protocol registration and search strategy

This review follows the recommendations established by the Preferred Reporting Items for Systematic Reviews and Meta-Analyses (PRISMA) statement [9]; it has been registered as a protocol in the International Prospective Register of Systematic Reviews (PROSPERO; https://www.crd.york.ac.uk) database on ID no CRD42020181539.

This review was conducted to estimate the pooled adverse complications and effects of age on each complication among COVID-19 patients. Potential studies were identified using databases PubMed/MEDLINE, Hinari, Google Scholar, and Google Search. Searches limited to English language and studies published from December 31, 2019, to April 10, 2020. Searching MeSH headings “2019-nCoV infection” OR “2019 novel coronavirus” OR COVID-19 OR “SARS-CoV-2” OR “2019-nCoV” AND “complication*” OR “Clinical characteristics” OR “Clinical course” NOT Animals were used to identify potential studies (additional Table).

### Eligibility identification

All studies which have a report on the mean age of the respondents, all age groups, and all PCR-confirmed COVID-19 cases; studies having a report on clinical complications; and studies conducted worldwide were included for this review. Retrospective and prospective observational studies, case series, and cross-sectional chart review study designs were included. Case report and qualitative studies were excluded from this review.

### Measurement of the outcome variables

The primary outcome of interest for this review was to estimate the pooled incidence of acute complications of COVID-19 patients. The second objective was to estimate the pooled effects of age on the incidence of COVID-19 complications. COVID-19 complications were declared as having ARDS, AKI, ACI, shock, and ALI among COVID-19 patients during hospitalization.

### Study selection and data collection

**Fig. 1:**
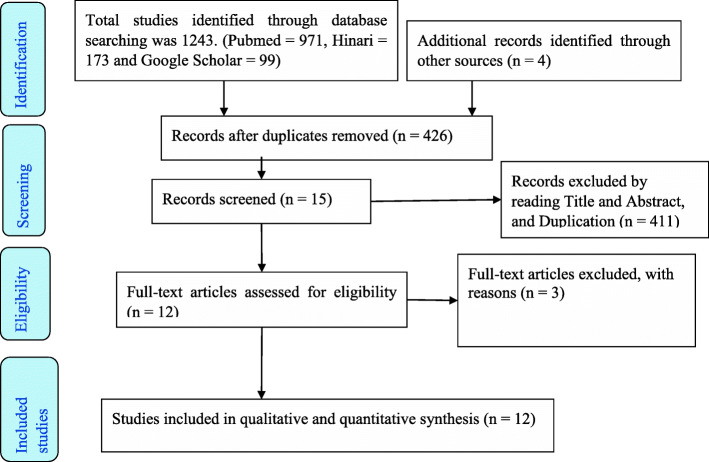
PRISMA flow diagram for included studies for COVID-19 patients

A total of 1247 articles were identified through the database search. After eligibility identification, 12 potential studies had been included for this qualitative and quantitative synthesis as summarized in the PRISMA flow diagram [9] (Fig. 1).

All studies were exported and managed using the Endnote™ version X9.2 (Thomson Reuters, Philadelphia, PA, USA) software. All duplicated studies were removed, and full-text articles searched using the Endnote software manually. Screening for eligibility of the individual articles was assessed independently by two reviewers (SA and MA) through review from the title, abstract, and full text. Exaggerated differences from the two reviewers narrowed through discussion and other reviewer members (ZT and DA).

### Quality assessment of individual studies

The quality of the included studies assessed using the critical appraisal tool to assess the quality of cross-sectional studies (AXIS) [10]. Two reviewers (SA and MA) independently assessed the quality of the included studies. The exaggerated discrepancy between the two reviewers was managed through discussion; otherwise, a third reviewer available to arbitrate any issues that remained unresolved (DA and ZT). The quality assessment tool measures a total of twenty questions. Overall scores of the AXIS tool ranges from 0 to 20 scores [10]. The highest score was a low risk of bias whereas the lowest score was a high risk of bias among the included studies.

### Data extraction and management

The data extraction for this review was summarized by two authors (SA and MA) using Microsoft Excel. The discrepancies between the two authors were managed through discussion between them and/or the other authors (DA and ZT). The main categories of data extraction were thematized on the main characteristics of the study which were author, year, journal, country, sample size, mean age, and sex; clinical characteristics (fever, cough, shortness of breath, myalgia and fatigue, headache, and diarrhea); clinical complications during hospitalization (ARDS, AKI, ACI, ALI, and shock); and imaging results.

### Qualitative and quantitative synthesis

The extracted data were imported to the STATA/MP version 16.0 software for further analysis. The descriptive analysis of the included articles was summarized using tables. The pooled estimate of each complication of COVID-19 patients was estimated by the random-effects model using DerSimonian-Laird model weight [11]. Heterogeneity in meta-analysis is mostly inevitable due to the differences in study quality, sample size, method, and different outcomes measured across studies [12, 13]. Statistical heterogeneity was checked by the Cochrane *Q* test and *I*^2^ statistics [14]. Sensitivity analysis was also conducted to determine the effect of single studies on the pooled estimates. Univariate meta-regression was conducted for each complication by the mean age of the respondent from primary studies using the random-effects model. Egger’s statistical test was used to check publication bias [15]. Statistically significant (*P* value < 0.05) Egger’s test indicates that the presence of a small study effect was handled using non-parametric trim and fill analysis using the random-effects model [16].

## Results

### Characteristics of included studies

All studies included for this systematic review and meta-analysis were conducted by Chinese scholars in China. The total sample size of 3064 COVID-19 patients was included in this review. The minimum and maximum sample sizes were 41 [17] and 1099 [18], respectively. The mean age of the patients from the included studies ranges from 47 [18] to 69 [19] years old (Table 1).

**Table 1:** Characteristics of the included studies for this review among COVID-19 patients

S. no	First author	Publication year	Country	Sample size	Mean age	Quality score^a^
1	Jin et al. [20]	2020	China	651	51	18
2	Chen et al. [21]	2020	China	99	55.5	19
3	Yang et al. [22]	2020	China	52	59.7	15
4	Zhang et al. [23]	2020	China	221	55	19
5	Cao et al. [24]	2020	China	102	54	19
6	Huang et al. [17]	2020	China	41	49	19
7	Du et al. [25]	2020	China	85	66	14
8	Wang et al. [26]	2020	China	138	56	18
9	Zhao et al. [27]	2020	China	77	52	17
10	Guan et al. [18]	2020	China	1099	47	19
11	Chen et al. [28]	2020	China	274	68	19
12	Deng et al. [19]	2019	China	225	69	17

^a^Quality score ranges from 0 to 20

### The pooled incidence of acute complication of novel coronavirus

**Fig. 2:**
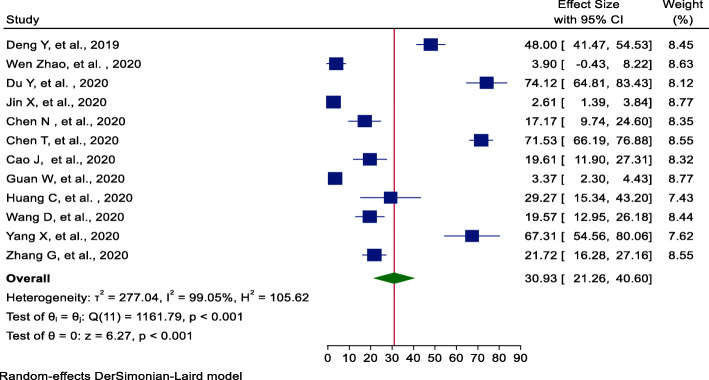
Forest plot of pooled incidence of ARDS among hospitalized COVID-19 patients

From twelve studies [17–28], the incidence of ARDS pooled estimate was 31% (95%CI = 21.26, 41.60); significant heterogeneity was observed among studies (*I*^2^ = 99.05, *P* value < 0.001). The highest weight among studies was observed from the studies conducted by Jin et al. and Guan et al. [18, 20] (Fig. 2). A small study effect (publication bias) was investigated using Egger’s statistical test [29]. Egger’s statistical test provides that there is no evidence for the presence of publication bias (*β* = −6.06, *P* value = 0.38).

**Fig. 3:**
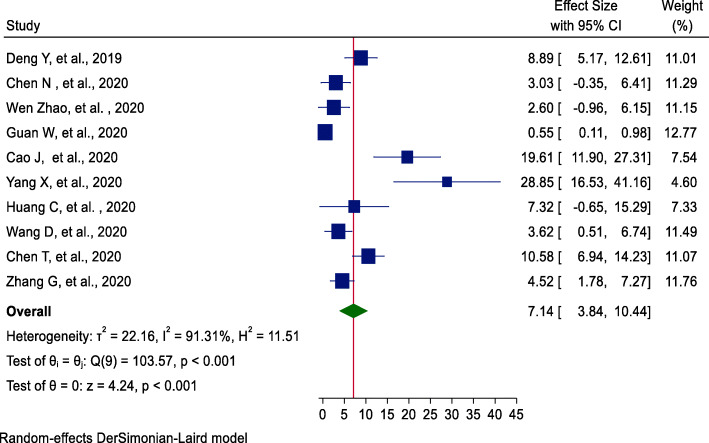
Forest plot of pooled incidence of AKI among hospitalized COVID-19 patients

Among ten studies [17–19, 21–24, 26–28], using the random-effects model, the incidence of AKI pooled estimate was 7% (95%CI = 3.84, 10.44). The highest weight among studies was observed from studies conducted by Guan et al. [11] (Fig. 3). Egger’s statistical test evidenced that there is no publication bias among the included studies (*β* = −4.31, *P* value = 0.13).

**Fig. 4:**
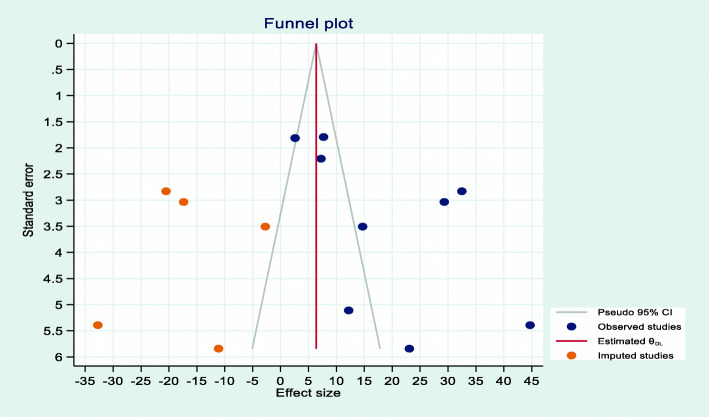
Trim and fill plot of pooled incidence of ACI among hospitalized COVID-19 patients

From nine studies [17, 19, 22–28], the incidence of AKI (18.89, 95%CI = 10.45, 27.29) had evidence of publication bias (*β* = −5.2, *P* value = 0.038). After random effects model trim and fill analysis (Fig. 4), the pooled incidence of ACI was 6.38 (95%CI = −2.84, 15.60) among hospitalized COVID-19 patients.

**Fig. 5:**
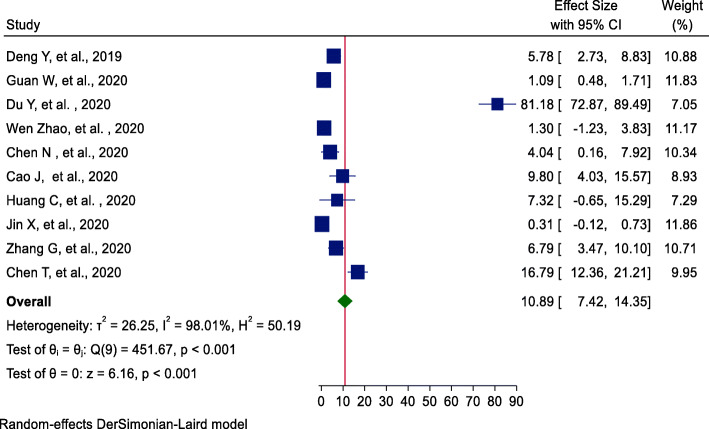
Forest plot of pooled incidence of shock among hospitalized COVID-19 patients

Using ten studies [17–21, 23–25, 27, 28], the incidence of shock among COVID-19 patients was 7% (95%CI = 3.84, 10.44). The highest weight among studies was observed from studies conducted by Jin et al. [20] (Fig. 5). Egger’s statistical test evidenced that there is no publication bias among the included studies (*β* = −4.51, *P* value = 0.29).

**Fig. 6:**
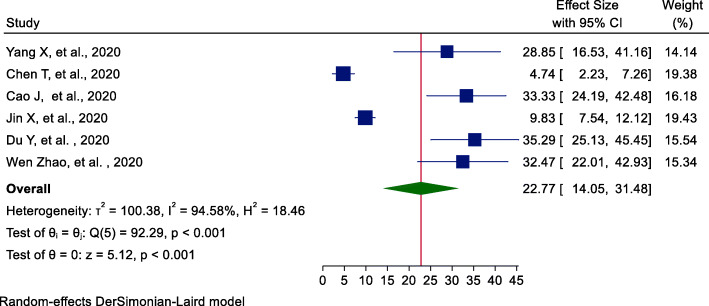
Forest plot of pooled incidence of ALI among hospitalized COVID-19 patients

Furthermore, the pooled incidence of ALI among COVID-19 patients was estimated using six studies [20, 22, 24, 25, 27, 28]. From these studies, the incidence of ALI among hospitalized COVID-19 patients was 22.77% (95%CI = 14.05, 31.48) (Fig. 6). Egger’s statistical test revealed that there is no publication bias among the included studies (*β* = 5.98, *P* value = 0.394). The pooled incidence of acute complication of COVID-19 is summarized and shown in Table 2.

**Table 2:** The pooled estimates of novel coronavirus complication among COVID-19 patients

Complication of COVID-19	No. of studies	Sample size (*N*)	Pooled incidence (95%CI)	Heterogeneity (*I*^2^, *P* value)	Egger’s *P* value
ARDS	12	3064	30.93 (21.26, 40.60)	99.05%, < 0.001	0.38
AKI	10	2328	7.14 (3.84, 10.44)	91.31%, < 0.001	0.13
ACI	14^a^	1215	6.38 (−2.84, 15.60)	95.08%, < 0.001	0.038
Shock	10	2874	10.89 (7.42, 14.35)	98.01%, < 0.001	0.29
ALI	6	1823	22.77 (14.05, 31.48)	94.58%, < 0.001	0.39

^a^After trim and fill analysis imputed five studies

### Meta-regression analysis of acute complications of COVID-19 patients

**Fig. 7:**
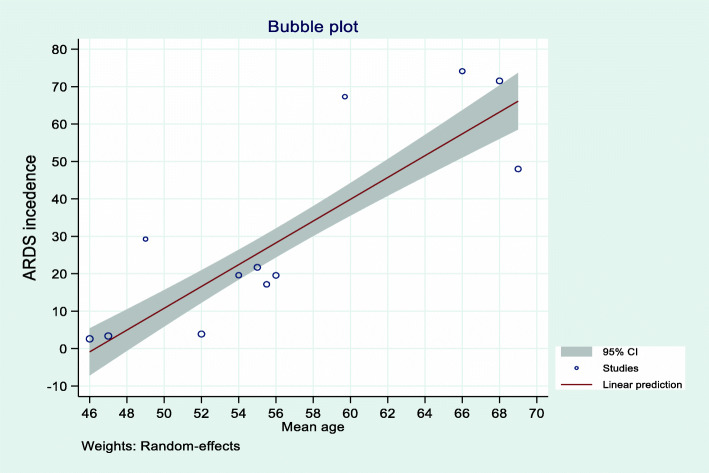
The bubble plot of the mean age and ARDS complication among COVID-19 patients

Univariate meta-regression analysis was conducted using the aggregated mean age of study level variables using the random-effects model. This meta-regression analysis evidenced that the mean age and the incidence of acute complication of 2019 novel coronavirus patients are statistically significant. As shown in the bubble plot (Fig. 7), the mean age and incidence of ARDS had a linear relationship. As the mean age increased by 1 year, the incidence of developing ARDS complications among COVID-19 patients would increase by a factor of 2.9 (*β* = 2.9, 95%CI 2.4–3.4) times, with a total incidence of ARDS complication of hospitalized COVID-19 patients explained by the covariate mean age of 88% (*R*^2^ = 87.62). Besides, the mean age and incidence of ACI among hospitalized COVID-19 patients had a linear relationship. The incidence of ACI complications among COVID-19 patients increased by a factor of 1.6 (*β* = 1.63, 95%CI 1.1–2.2) times as the mean age increased by 1 year with model adjusted *R*^2^ of 85%. The mean age and incidence of AKI had a statistically significant linear relationship. The likelihood of developing AKI would increase by a factor of 0.4 (*β* = 0.4, 95%CI 0.04–0.72) times as the mean age increased by 1 year, with an incidence of AKI explained by the mean age of 54% (*R*^2^ = 54.07) (Table 3).

**Table 3:** Meta-regression analysis effects of the mean age on acute complications of COVID-19 patients

Acute complication of COVID-19 patients during hospitalization	Study level variable mean age of the hospitalized patients			
	Adjusted *R*^2^	Standard error	Beta (*β*) (95%CI)	*P* value
ARDS	87.62	0.25	2.91 (2.41, 3.41)	< 0.001
ACI	84.63	0.29	1.63 (1.06, 2.19)	< 0.001
AKI	54.07	0.17	0.38 (0.04, 0.72)	0.026
ALI	0.00	1.02	−0.04 (−2.05, 1.97)	0.97
Shock	25.97	0.19	1.13 (0.76, 1.50)	< 0.001

## Discussion

In the past 4 months, more than 2.8 million individuals have been infected by COVID-19 coronavirus [7]. The virus was an emerging public health challenge around the globe. Currently, the virus has effective vaccination evidenced by different studies [30, 31]. Therefore, to date, social distancing, staying home, frequent hand washing for at least 20 s, and avoiding handshaking have been put an option to prevent the transmission. Clinical complications and death were higher among old populations.

This review revealed that 31% of the patients who experienced ARDS followed by ALI, shock, AKI, and ACI experienced 23%, 11%, 7%, and 6% of the cases, respectively. From the report of four studies [19, 21, 22, 24], in patients with ARDS, AKI, and ACI complications, 75%, 91%, and 86% of them died, respectively. Therefore, early identification of complications and critical follow-up and treatment was needed for COVID-19 patients.

The incidence of ARDS and ALI was similar to previous reports among laboratory-confirmed MERS-CoV infection (40% and 31%, respectively) in Saudi Arabia [32]. Besides, the incidence of AKI was lower than a study from Saudi Arabia among laboratory-confirmed MERS-CoV patients [32]. ARDS and shock incidence were higher than the severe influenza season in China during 2017–2018 [33]. On the other hand, ARDS was lower than a report from the Middle East respiratory syndrome coronavirus (MERS-CoV) infection hospitalized patients in China (88%) whereas shock frequency was similar with MERS-CoV [34].

From a univariate meta-regression analysis, age had a linear relationship with ARDS, AKI, ACI, and shock complications among COVID-19 hospitalized patients. As the mean age increased by 1 year, the incidence of ARDS, ACI, AKI, and shock increased by a factor of 2.9, 1.6, 0.4, and 1.1 times, respectively. The finding was supported by previous studies conducted among MERS-CoV patients in China, which is being older has a higher risk of mortality [34]. Another study in Saudi Arabia evidenced that the likelihood of death increased by four times among hospitalized MERS-CoV patients younger than 65 years [32]. Besides, the hazard of severe acute respiratory syndrome (SARS)-related mortality increased by 3.5 times in those with age above 60 years among hospitalized patients [35]. The possible justification for the risk of developing complications and death among older patients will be due to prevalent chronic comorbidity among older patients.

The result of this systematic review and meta-analysis highlights the clinical complications of COVID-19 patients. Also, this review documents the association between age and complications among COVID-19 patients during hospitalization. Evaluating these results will help clinicians and other stakeholders to mitigate the progression of the infection.

### Strength and limitation of the review

This study was identified as the pooled evidence of adverse clinical complications of COVID-19. Besides, the study shows the association between age and each adverse complication used to timely interventions. The first limitation of this review was few studies were included for the evaluation of adverse complications and no studies were included outside China. Second, substantial statistically significant heterogeneity was observed across studies that undermine the pooled estimate of COVID-19 complications which suggests that chance could be responsible for between-study variability. These heterogeneities could be due to different study designs, sample size variations, and age variations among the included study participants. Since all studies were from China, it will limit the generalizability to other contents.

## Conclusion and recommendation

The novel 2019 coronavirus causes significant morbidity and mortality associated with multiorgan failure especially among older individuals. The review summarizes the pooled complications of ARDS, AKI, ACI, ALI, and shock. Being aged had a risk of developing adverse complications of COVID-19 during hospitalization. Since older age had more risk of developing complications and a high risk of mortality, health care providers should take care of primary attention for those groups.

## Supplementary Information

**Additional file 1: Table S1**. Search strategies and entry terms for novel coronavirus (COVID-19).

## Data Availability

All data is available in the manuscript.

## Abbreviations

ARDS: Acute respiratory distress syndrome
AKI: Acute kidney injury
ACI: Acute cardiac injury
ALI: Acute liver injury
MERS-CoV: Middle East respiratory syndrome coronavirus

## References

[1] Coronaviruses NI. National Institute of Allergy and Infectious Diseases. NIH Natl. Institue Allergy Infect. Dis. NIAID https://www.niaid.nih.gov/diseases-conditions/coronaviruses. Accessed 23 Jul 2020.

[2] Ward MP, Li X, Tian K. Novel coronavirus 2019, an emerging public health emergency. Transbound Emerg Dis. 2020;67(2):469. 10.1111/tbed.13509 PMC7168532 32077206

[3] Gutiérrez-ocampo E, Villamizar-peña R, Holguin-rivera Y, Franco-paredes C, Henao-martinez AF, Paniz-mondolfi A, et al. Clinical laboratory and imaging features of COVID-19: a systematic review and meta-analysis. Travel Med Infect Dis. 2020:101623. 10.1016/j.tmaid.2020.101623. 10.1016/j.tmaid.2020.101623 PMC7102608 32179124

[4] Guan W, Ni Z, Hu Y, Liang W, Ou C, He J, Liu L, Shan H, Lei CL, Hui DSC, du B, Li LJ, Zeng G, Yuen KY, Chen RC, Tang CL, Wang T, Chen PY, Xiang J, Li SY, Wang JL, Liang ZJ, Peng YX, Wei L, Liu Y, Hu YH, Peng P, Wang JM, Liu JY, Chen Z, Li G, Zheng ZJ, Qiu SQ, Luo J, Ye CJ, Zhu SY, Zhong NS, China Medical Treatment Expert Group for Covid-19 (2020). Clinical characteristics of coronavirus disease 2019 in China. N Engl J Med..

[5] Yang J, Zheng Y, Gou X, Pu K, Chen Z, Guo Q, Ji R, Wang H, Wang Y, Zhou Y (2020). Prevalence of comorbidities and its effects in coronavirus disease 2019 patients: a systematic review and meta-analysis. Int J Infect Dis..

[6] Adhikari SP, Meng S, Wu Y, Mao Y, Ye R, Wang Q (2020). Epidemiology, causes, clinical manifestation and diagnosis, prevention and control of coronavirus disease (COVID-19) during the early outbreak period: a scoping review.

[7] World Health Organization (WHO) (2020). Coronavirus disease 2019 (COVID-19) situation report – 97.

[8] Yang X, Yu Y, Xu J, Shu H, Xia J, Liu H (2020). Clinical course and outcomes of critically ill patients with SARS-CoV-2 pneumonia in Wuhan, China: a single-centered, retrospective, observational study. Lancet Respir Med.

[9] Moher D, Liberati A, Tetzlaff J, Altman DG, Altman D, Antes G (2009). Preferred Reporting Items for Systematic Reviews and Meta-Analyses: the PRISMA statement. Plos Med..

[10] Downes MJ, Brennan ML, Williams HC, Dean RS (2016). Development of a critical appraisal tool to assess the quality of cross-sectional studies (AXIS). BMJ Open..

[11] DerSimonian R, Laird N (1986). Meta-analysis in clinical trials. Control Clin Trials..

[12] Huedo-Medina TB, Sánchez-Meca J, Marín-Martínez F, Botella J (2006). Assessing heterogeneity in meta-analysis: Q statistic or I 2 Index?. Psychol Methods..

[13] Thompson SG, Smith TC, Sharp SJ (1997). Investigating underlying risk as a source of heterogeneity in meta-analysis. Stat Med..

[14] Green JP, Cochrane S. Handbook for systematic reviews of interventions. Cochrane Collaboration and John Wiley & Sons Ltd; 2017. p. 1-674.

[15] Egger M, Smith GD, Schneider M, Minder C (1997). Bias in meta-analysis detected by a simple, graphical test. Br Med J..

[16] Duval S, Tweedie R (2000). A nonparametric “trim and fill” method of accounting for publication bias in meta-analysis. J Am Stat Assoc.

[17] Huang C, Wang Y, Li X, Ren L, Zhao J, Hu Y, Zhang L, Fan G, Xu J, Gu X, Cheng Z, Yu T, Xia J, Wei Y, Wu W, Xie X, Yin W, Li H, Liu M, Xiao Y, Gao H, Guo L, Xie J, Wang G, Jiang R, Gao Z, Jin Q, Wang J, Cao B (2020). Clinical features of patients infected with 2019 novel coronavirus in Wuhan,China. Lancet..

[18] Guan W, Ni Z, Hu YYYY, Liang W, Ou C, He J (2020). Clinical characteristics of coronavirus disease 2019 in China. N Engl J Med.

[19] Deng Y, Liu W, Liu K, Fang Y, Shang J, Zhou L (2020). Clinical characteristics of fatal and recovered cases of coronavirus disease 2019 (COVID-19) in Wuhan, China. Chin Med J (Engl).

[20] Jin X, Lian JS, Hu JH, Gao J, Zheng L, Zhang YM, Hao SR, Jia HY, Cai H, Zhang XL, Yu GD. Epidemiological, clinical and virological characteristics of 74 cases of coronavirus-infected disease 2019 (COVID-19) with gastrointestinal symptoms. Gut. 2020;69(6):1002–9. 10.1136/gutjnl-2020-320926 PMC7133387 32213556

[21] Chen N, Zhou M, Dong X, Qu J, Gong F, Han Y, Qiu Y, Wang J, Liu Y, Wei Y, Xia J', Yu T, Zhang X, Zhang L (2020). Epidemiological and clinical characteristics of 99 cases of 2019 novel coronavirus pneumonia in Wuhan, China: a descriptive study. Lancet.

[22] Yang X, Yu Y, Xu J, Shu H, Xia J, Liu H (2020). Clinical course and outcomes of critically ill patients with SARS-CoV-2 pneumonia in Wuhan, China: a single-centered, retrospective, observational study. Lancet Respir Med.

[23] Zhang G, Hu C, Luo L, Fang F, Chen Y, Li J, Peng Z, Pan H. Clinical features and outcomes of 221 patients with COVID-19 in Wuhan, China. MedRxiv. 2020. 10.1016/j.jcv.2020.104364 PMC7194884 32311650

[24] Cao J, Tu WJ, Cheng W, Yu L, Liu YK, Hu X, Liu Q. Clinical features and short-term outcomes of 102 patients with coronavirus disease 2019 in Wuhan, China. Clin Infect Dis. 2020;71(15):748–55. 10.1093/cid/ciaa243 PMC7184479 32239127

[25] Du Y, Tu L, Zhu P, Mu M, Wang R, Yang P, Wang X, Hu C, Ping R, Hu P, Li T. Clinical features of 85 fatal cases of COVID-19 from Wuhan. A retrospective observational study. Am J Respir Crit Care Med. 2020;201(11):1372–9. 10.1164/rccm.202003-0543OC PMC7258652 32242738

[26] Wang D, Hu B, Hu C, Zhu F, Liu X, Zhang J, Wang B, Xiang H, Cheng Z, Xiong Y, Zhao Y, Li Y, Wang X, Peng Z (2020). Clinical characteristics of 138 hospitalized patients with 2019 novel coronavirus-infected pneumonia in Wuhan, China. JAMA.

[27] Zhao W, Yu S, Zha X, Wang N, Pang Q, Dongzeng Li AL. Clinical characteristics and durations of hospitalized patients with COVID-19 in Beijing: a retrospective cohort study Wen. medRxiv. 2020. 10.1101/2020.03.13.20035436.

[28] Chen T, Wu D, Chen H, Yan W, Yang D, Chen G (2020). Clinical characteristics of 113 deceased patients with coronavirus disease 2019: retrospective study. BMJ..

[29] Irwig L, Macaskill P, Berry G, Glasziou P (1998). Bias in meta-analysis detected by a simple, graphical test. Graphical test is itself biased. BMJ..

[30] Folegatti PM, Ewer KJ, Aley PK, Angus B, Becker S, Belij-Rammerstorfer S (2020). Safety and immunogenicity of the ChAdOx1 nCoV-19 vaccine against SARS-CoV-2: a preliminary report of a phase 1/2, single-blind, randomised controlled trial. Lancet.

[31] Sahin U, Muik A, Derhovanessian E, Vogler I, Kranz LM (2020). Concurrent human antibody and T H 1 type T-cell responses elicited by a COVID-19 RNA vaccine.

[32] Saad M, Omrani AS, Baig K, Bahloul A, Elzein F, Abdul M (2014). International Journal of Infectious Diseases Clinical aspects and outcomes of 70 patients with Middle East respiratory syndrome coronavirus infection: a single-center experience in Saudi Arabia. Int J Infect Dis.

[33] Fu X, Zhou Y, Wu J, Liu X, Ding C, Huang C (2019). Clinical characteristics and outcomes during a severe influenza season in China during 2017-2018. BMC Infect Dis.

[34] Habib AMG, Ali MAE, Zouaoui BR, Taha MAH, Mohammed BS, Saquib N (2019). Clinical outcomes among hospital patients with Middle East respiratory syndrome coronavirus (MERS-CoV) infection. BMC Infect Dis.

[35] Hu X, Deng Y, Wang J, Li H, Li M, Lu Z (2004). Short term outcome and risk factors for mortality in adults with critical severe acute respiratory syndrome (SARS). J Huazhong Univ Sci Technol Med Sci..

