# Supplementary material for: The effect of age on the incidence of COVID-19 complications: a systematic review and meta-analysis
Source: Syst Rev. 2021 Mar 20;10:80. doi: 10.1186/s13643-021-01636-2 (PMC7980104; doi:10.1186/s13643-021-01636-2)
Supplement: Supplementary file 1 — Additional file 1: Table S1. Search strategies and entry terms for novel coronavirus (COVID-19). [file 13643_2021_1636_MOESM1_ESM.docx]

Table 1: Search strategies and entry terms for novel coronavirus (COVID-19)

| PubMed/MEDLINE database, Time period search for the last one year | | | | |
| --- | --- | --- | --- | --- |
| MeSH Heading | Entry term (Synonyms) | Combination | Articles | Date of search |
| novel coronavirus | - 2019-nCoV infection - coronavirus disease 2019 - COVID-19 pandemic - 2019-nCoV disease - 2019 novel coronavirus disease - COVID19 - 2019 novel coronavirus infection - Wuhan coronavirus - Wuhan seafood market pneumonia virus - COVID19 virus - COVID-19 virus - coronavirus disease 2019 virus - SARS-CoV-2 - 2019-nCoV - 2019 novel coronavirus | (novel coronavirus[Title/Abstract]) OR (2019-nCoV infection[Title/Abstract]) OR (coronavirus disease 2019[Title/Abstract]) OR (COVID-19 pandemic[Title/Abstract]) OR (2019-nCoV disease[Title/Abstract]) OR (2019 novel coronavirus disease[Title/Abstract]) OR (COVID19[Title/Abstract]) OR (2019 novel coronavirus infection[Title/Abstract]) OR (Wuhan coronavirus[Title/Abstract]) OR (Wuhan seafood market pneumonia virus[Title/Abstract]) OR (COVID19 virus[Title/Abstract]) OR (COVID-19 virus[Title/Abstract]) OR (coronavirus disease 2019 virus[Title/Abstract]) OR (SARS-CoV-2[Title/Abstract]) OR (2019-nCoV[Title/Abstract]) OR (2019 novel coronavirus[Title/Abstract]) | 453 | 4/3/2020 |
| Hinari database, time period of search 12/1/2019 to 4/2/2020 | | | | |
| novel coronavirus | - 2019-nCoV infection - coronavirus disease 2019 - COVID-19 pandemic - 2019-nCoV disease - 2019 novel coronavirus disease - COVID19 - 2019 novel coronavirus infection - Wuhan coronavirus - Wuhan seafood market pneumonia virus - COVID19 virus - COVID-19 virus - coronavirus disease 2019 virus - SARS-CoV-2 - 2019-nCoV - 2019 novel coronavirus | ((TitleCombined:("2019-nCoV infection")) OR (TitleCombined:("2019-nCoV")) OR (TitleCombined:("coronavirus disease 2019")) OR (TitleCombined:("SARS-CoV-2")) OR (TitleCombined:("coronavirus disease 2019 virus")) OR (TitleCombined:("COVID-19 pandemic")) OR (TitleCombined:("COVID-19 virus")) OR (TitleCombined:("COVID19 ")) OR (TitleCombined:("2019-nCoV disease")) OR (TitleCombined:("COVID19 virus")) OR (TitleCombined:("2019 novel coronavirus")) OR (TitleCombined:("Wuhan seafood market pneumonia virus")) OR (TitleCombined:("Wuhan coronavirus")) OR (TitleCombined:("2019 novel coronavirus infection")) OR (TitleCombined:("2019 novel coronavirus disease ") | 129 | 4/3/2020  /03/2019 |
